# A Study of the Improved A* Algorithm Incorporating Road Factors for Path Planning in Off-Road Emergency Rescue Scenarios

**DOI:** 10.3390/s24175643

**Published:** 2024-08-30

**Authors:** Dequan Zhao, Li Ni, Kefa Zhou, Zhihong Lv, Guangjun Qu, Yue Gao, Weiting Yuan, Qiulan Wu, Feng Zhang, Qing Zhang

**Affiliations:** 1 School of Information Science & Engineering, Shandong Agricultural University, Tai’an 271018, China; 2022110551@sdau.edu.cn (D.Z.); 2022110547@sdau.edu.cn (Y.G.); zxylsg@sdau.edu.cn (Q.W.); 2 Institute of Aerospace Information Innovation, Chinese Academy of Sciences, Beijing 100094, China; nili@aircas.ac.cn; 3 Technology and Engineering Center for Space Utilization, Chinese Academy of Sciences, Beijing 100045, China; zhoukf@csu.ac.cn; 4 School of Marine Technology and Geomatics, Jiangsu Ocean University, Lianyungang 222005, China; 2022220228@jou.edu.cn (Z.L.); 13233820238@163.com (W.Y.); 5 School of Mechanical and Electrical Engineering, Beijing University of Chemical Technology, Beijing 100020, China; 2022210351@buct.edu.cn

**Keywords:** path planning, A-star algorithm, off-road vehicles, efficiency, emergency rescue

## Abstract

To address the problem of ignoring unpaved roads when planning off-road emergency rescue paths, an improved A* algorithm that incorporates road factors is developed to create an off-road emergency rescue path planning model in this study. To reduce the number of search nodes and improve the efficiency of path searches, the current node is classified according to the angle between the line connecting the node and the target point and the due east direction. Additionally, the search direction is determined in real time through an optimization method to improve the path search efficiency. To identify the path with the shortest travel time suitable for emergency rescue in wilderness scenarios, a heuristic function based on the fusion of road factors and a path planning model for off-road emergency rescue is developed, and the characteristics of existing roads are weighted in the process of path searching to bias the selection process toward unpaved roads with high accessibility. The experiments show that the improved A* algorithm significantly reduces the travel time of off-road vehicles and that path selection is enhanced compared to that with the traditional A* algorithm; moreover, the improved A* algorithm reduces the number of nodes by 16.784% and improves the search efficiency by 27.18% compared with the traditional 16-direction search method. The simulation results indicate that the improved algorithm reduces the travel time of off-road vehicles by 21.298% and improves the search efficiency by 93.901% compared to the traditional A* algorithm, thus greatly enhancing off-road path planning.

## 1. Introduction

Off-road autonomous ground vehicles (AGVs) have attracted widespread attention in the fields of field exploration, disaster relief, military operations, etc. [1,2,3]. Especially in emergency rescue missions, off-road AGVs are widely used due to their off-road accessibility and ability to quickly reach the target point. The off-road environment is characterized by large terrain fluctuations and complex surface types, which cause delays and present obstacles for the passage of off-road AGVs [4]. Among the various complex road types in wilderness areas, unpaved roads with high capacities due to long-term vehicle traffic are common. However, the existing off-road AGV path planning algorithms often ignore the fact that these unpaved roads are more accessible than many other off-road paths. Therefore, it is highly important to establish a path planning method that considers the risks of off-road scenarios and integrates existing dirt roads into the simulation environment to complete rescue or exploration tasks in off-road cases.

The existing path planning methods are predominantly designed for Indoor, urban, and other two-dimensional structured environments. However, off-road scenarios consist of complex environments composed of various terrain types, and therefore, the current path planning constraints are often unsuitable for unstructured off-road settings [5,6]. Path planning algorithms in off-road environments face the following challenges: variable terrain slopes that severely limit or affect off-road vehicle access and complex soil types that are easily influenced by weather [7,8,9,10]. Additionally, most current planning methods focus on structured environments, and thus, researchers have proposed several improved planning methods for off-road cases based on traditional path planning algorithms. For example, to assess the combined effects of slope and surface attributes, an improved A* algorithm combined with the moving window method was proposed, enabling robots to effectively avoid obstacles [11]. Jiang et al. [12] introduced a global path planning method based on the Rapidly-exploring Random Trees Star algorithm (RRT*) to generate high-confidence global paths in off-road conditions, with terrain elevation uncertainty considered in the cost function. Ye et al. proposed an improved motion-constrained bidirectional RRT (IKB-RRT) path planning algorithm. The algorithm aims to quickly design an effective and collision-free path to facilitate the autonomous navigation of mobile robots in orchard environments characterized by irregular obstacle distribution [13,14]. In terms of global path smoothing, geometric curve methods, such as the b-spline curve and quintic polynomial curve methods, transform the path planning problem into a two-point boundary value problem [15,16]. To study the impact of soil deformation on vehicle movement, Gonzalez et al. [4] proposed a path planning method that divides terrain into GO/NOGO regions based on the soil cone index (CI). Notably, this method has been integrated into the next-generation NATO Reference Mobility Model (NG-NRMM). In parallel to the work on the NG-NRMM, several simulation models have been developed. For instance, Xia [17] used the finite element method (FEM) to establish a tire-terrain interaction model to predict tire mobility. Existing studies on the impact of soil on off-road vehicle traversal required measurements of numerous parameters. Path planning methods that divide GO/NOGO regions need to compare the soil CI with the vehicle cone index (VCI), where the VCI values are related to the vehicle mobility index (MI). Moreover, the MI is associated with factors such as the ground pressure coefficient, axle load coefficient, tire coefficient, antislip coefficient, engine coefficient, and transmission coefficient. Notably, quantitative calculations of soil softness and friction involve many parameters and overly complex processes.

In recent years, researchers have conducted exploratory studies involving off-road path planning, with the key objective of generating safe and feasible paths from an initial position to a specified target based on given off-road environment information [18,19,20]. Specifically, optimizing path search algorithms has played a crucial role in ensuring the effectiveness of off-road vehicle missions and meeting travel time, fuel consumption, and risk avoidance requirements [21,22]. Path planning is generally categorized into global path planning and local path planning according to the degree to which environmental information is considered; notably, global path planning is based on a priori complete information, and local path planning is based on sensor information [23,24,25]. From the perspective of obtaining obstacle information, global path planning is a type of static planning, while local path planning is a type of dynamic planning using real-time environmental data as inputs [26,27]. Some of the commonly used methods in global path planning algorithms include evolutionary algorithms, Dijkstra’s algorithm, and the A* algorithm [28,29]. Evolutionary algorithms are often considered optimal for local use but incur high computational costs. While Dijkstra’s algorithm can be used to find the shortest path, its search efficiency is low due to excessive searching. The A* algorithm is one of the most effective direct search methods for finding the shortest path in static road networks and is widely used in global path planning due to its efficiency and optimality. However, the A* algorithm requires traversing current nodes and selecting the lowest-cost node during the search process. When the search environment is large, the algorithm may require long computational times due to significant recursion depth issues or an abundance of search nodes. Thus, the main challenges associated with the A* algorithm are Improving Its global search capability and convergence speed [30].

To address the above problems, many scholars around the world have optimized the A* algorithm from multiple dimensions, such as valuation functions and function constraints. Zhang et al. [31] introduced a time constraint factor to reduce the number of bends in the optimal path in the path search process. Cao et al. [32] adjusted the weight factor in the A* algorithm and significantly improved the search efficiency. Guruji [33] et al. reduced the computational time of the traditional A algorithm by 65% by optimizing the cost function, thus reducing the numbers of redundant points and inflection points in path planning and ensuring the real-time performance of the algorithm. In obstacle avoidance path planning, Duan et al. [34] introduced a safety cost matrix into the A* algorithm and corrected the cost function to ensure the selection of optimal safe paths in different environments. Tang et al. summarized the latest progress in autonomous obstacle avoidance technology and pointed out that planning performance can be improved by strategically integrating various algorithms and enhancing the intelligence of traditional algorithms [35]. However, with the increases in the spatial dimension and the complexity of features, the computational burden of the A* algorithm increases dramatically, which greatly reduces the efficiency of path planning; thus, there have been fewer studies of off-road path planning based on the A* algorithm over long distances and multiple terrains.

Although many scholars have made great progress in solving the above problems, most of the existing global path planning methods ignore the impact of existing roads on cross-country path planning. To solve this problem, the work performed in this study is as follows: (1) To effectively obtain a 3D model of an unknown off-road environment, a rasterization modeling method is proposed for off-road environment mapping and terrain reconstruction. The continuous terrain is divided into discrete grids, a recognizable off-road raster map is established, including slope and surface attribute information, and a traversability rating table is generated based on known information. (2) To evaluate vehicle traversability under different terrain conditions and intuitively reflect the influence of surface properties on off-road AGVs, the effects of surface friction and soil softness on traffic are evaluated, and changes in the speed of off-road vehicles under different ground conditions are considered when determining the influence of different ground objects and soil types on the travel of off-road vehicles. (3) In terms of the global path algorithm, an optimized A* algorithm that integrates outdoor road factors is introduced, a grid weight matrix is constructed, and a grid penalty coefficient is set to establish a path-finding algorithm for wilderness areas. Additionally, a heuristic function integrating distance factors and time factors is developed to improve the rationality of road searches in the path-finding algorithm. To reduce the time cost of path searches, the study area is divided based on the traditional 16-direction search method, and a dynamic search method is used to reduce the number of search nodes and travel time. Finally, the rationality of the proposed method is verified through simulation experiments.

The remaining sections of this paper are organized as follows. Section 2 introduces the selected test area for the study and details the process of 3D terrain reconstruction. In Section 3, the application of the A* algorithm in 3D environments is described, and the optimized A* algorithm is proposed. In Section 4, through simulation experiments, the effectiveness of the algorithm is verified, and the research results are presented. Section 5 presents the simulation results, and conclusions are drawn from the findings.

## 2. Three-Dimensional Terrain Surface Modeling

The characteristics of off-road scenarios that affect the useability of off-road vehicles include terrain, slope, slope direction, soil type, surface material, surface hardness, weather factors, vegetation, and hydrological conditions, among other factors [36]. In most cases, the main determinants of terrain accessibility are terrain elements (such as slope gradient and slope orientation) and surface attributes (such as soil type and surface material) [37]. Therefore, terrain elements and surface attributes are treated as the main risk factors in off-road scenarios, and terrain reconstruction and surface attribute processing are performed to construct 3D off-road raster maps that can characterize the main risk features in off-road scenarios.

### 2.1. Study Area and Dataset

**Figure 1:**
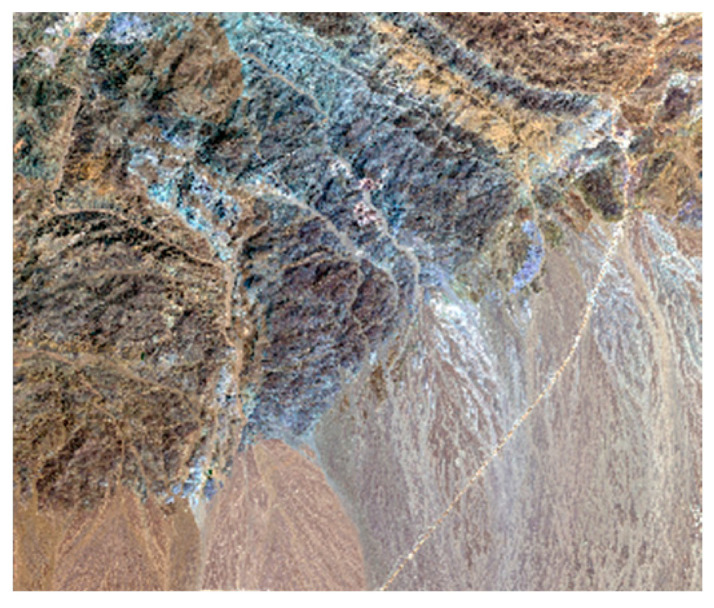
High-resolution image of the test area.

Considering the representativeness of the given off-road terrain, the test site selected for this study was in an area with distinct topographic features near Emin County, Xinjiang, with a specific latitude of 84° E and longitude of 46° N. The selected off-road area was approximately 80 km^2^. To increase the accuracy of the study, GaoFen II image data with a resolution of 1 m and DEM data with a resolution of 12.5 m, which are suitable for remote land classification, were used in this experiment. A high-resolution image of the experimental area is shown in Figure 1.

**Figure 2:**
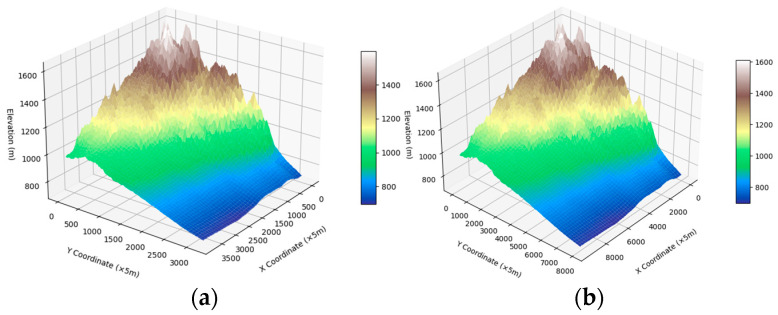
Topographic map at the original resolution and reconstructed topographic map: (**a**) original 12.5 m resolution topographic map and (**b**) reconstructed topographic map at a 5 m resolution.

In the path planning process, off-road vehicles are regarded as mass points in the raster, but the 30 m resolution digital elevation data provided in many public datasets (e.g., the GDEMV3, ASTER GDEM, and SRTMSLOPE datasets) may not be able to meet the needs of vehicle path planning researchers [38]. To obtain high-resolution DEM data, DEM data with a resolution of 12.5 m, as shown in Figure 2a, are selected, and the kriging interpolation method [19] is used to generate a DEM with a resolution of 5 m.

There are three main methods of kriging interpolation, depending on whether the mean of the data is known (simple kriging) or unknown (ordinary kriging and pan kriging). Ordinary kriging is the most common and widely used kriging method and is the default method; in pan kriging, it is assumed that there is overlap in the data, and polynomials are subtracted from the original measurement points. Moreover, autocorrelation is modeled considering random errors. After fitting the model considering random errors, the polynomials are added back to the model to produce new predictions. In geostatistics, the most commonly used method is the ordinary kriging method [39] because the mean is not known a priori, and only spatial variability is meaningful. Based on the ordinary kriging formula, a weighted combination of known terrain points is used to interpolate unknown elevations at other terrain points with the following expression:

(1) $$\hat{z}=\sum_{i=1}^{N}\omega_{i}z\left(s_{i}\right)$$

where $z(s_{i})$ is the measured value at the ith position. $\omega_{i}$ is the weighting value associated with terrain point $z(s_{i})$. The parameter *I* denotes the sample set. N is the total number of samples. In the anti-distance weight method, the weight *ω_i_* depends only on the distance of the forecasting position from a selected position. The minimum nonbiased and estimated variance is generally used as the standard for $\omega_{i}$. The general estimation variance in ordinary kriging is typically smaller than that in other kriging methods. After weights are set, the elevations at unknown positions can be estimated according to formula (1). An example of DEM reconstruction is shown in Figure 2b.

#### 2.1.1. Surface Slope Modeling

In off-road scenarios, the terrain slope is an important parameter affecting off-road vehicle path planning [40]. The slope refers to the rate of change in the elevation in a certain direction within a certain range, which can be obtained by determining the angle between the horizontal plane and the fitted plane and represents the degree of inclination of the tangent plane at a certain point. As the slope increases, surface adhesion decreases, and resistance increases. Therefore, the slope is the main factor affecting off-road traversability. The maximum change in elevation over the distance between a unit and its neighboring units is used to determine the steepest downslope for that unit; the lower the slope is, the flatter the terrain, and the higher the slope is, the steeper the terrain.

**Figure 3:**
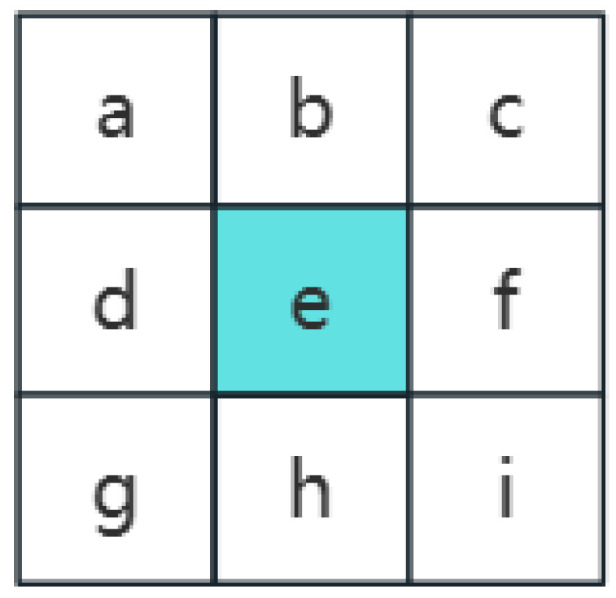
Slope grid, e is the center point of the window.

In this study, according to the method in the Slope Calculation Tool in ArcGIS, based on the 3 × 3 elevation data in Figure 3 and using the Sobel filter to estimate the gradient, the gradient estimation equations for the two directions (x and y) from the center point e of the window (shown below) are as follows:

(2) $$\frac{dz}{dx}=\frac{\left[\left(c+2f+i\right)*wght1\right]-\left[\left(a+2d+g\right)*wght2\right]}{8cellsize}$$

(3) $$\frac{dz}{dy}=\frac{\left[\left(g+2h+i\right)*wght3\right]-\left[\left(a+2d+c\right)*wght4\right]}{8cellsize}$$

where *wght*1 and towght4 are the weights based on whether the neighboring points have available data. For example, if a neighboring point has no data (No Data), then its weight is set to zero.

According to the automobile dynamics index, the maximum climbing angle of a vehicle is related to the maximum climbing ability of the vehicle. Generally, the climbing ability of a four-wheeled vehicle corresponds to approximately 16.7°. However, off-road vehicles are usually driven on bad roads or in roadless conditions, so their climbing ability is comparatively better, and their maximum climbing angle approximately 31° [41]. To ensure that areas are accessible to off-road vehicles, the uphill and downhill thresholds of vehicles are set to 31°. If the slope of a grid is not within this range, the grid is not passable, and vice versa [42]. The slope angle is divided into various levels, and each level is assigned a different slope weight factor, ranging from 0 to 1 [43]. In general, the harder it is for a vehicle to a climb a slope in a given interval, the lower the weight of the corresponding grid will be. The interval division scheme and weights for slopes are shown in Table 1.

**Figure 4:**
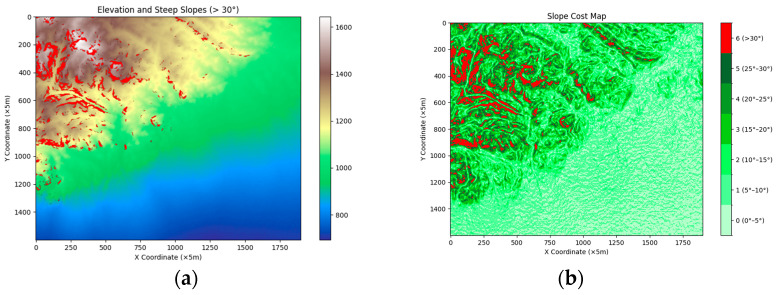
(**a**) Elevation map, where the red areas indicate areas with slopes greater than 30°; (**b**) map showing different slope classes.

Through the above elevation initialization process, the processed raster information is read in Python to obtain a slope class information map, as shown in Figure 4b.

#### 2.1.2. Surface Attribute Modeling

Considering that off-road vehicles are affected by surface attributes such as water conditions, soil quality, vegetation, and road types in off-road scenarios, the “Land supervision and classification” function in GIS PRO 3.0.0 software is utilized to process surface attributes by using the support vector machine (SVM) feature extraction method. A high-resolution remote sensing image of a given cross-country area in the study region mainly contains five surface attributes: bare land, sandy land, natural pastureland, saline land, and bare rocky gravel land.

**Figure 5:**
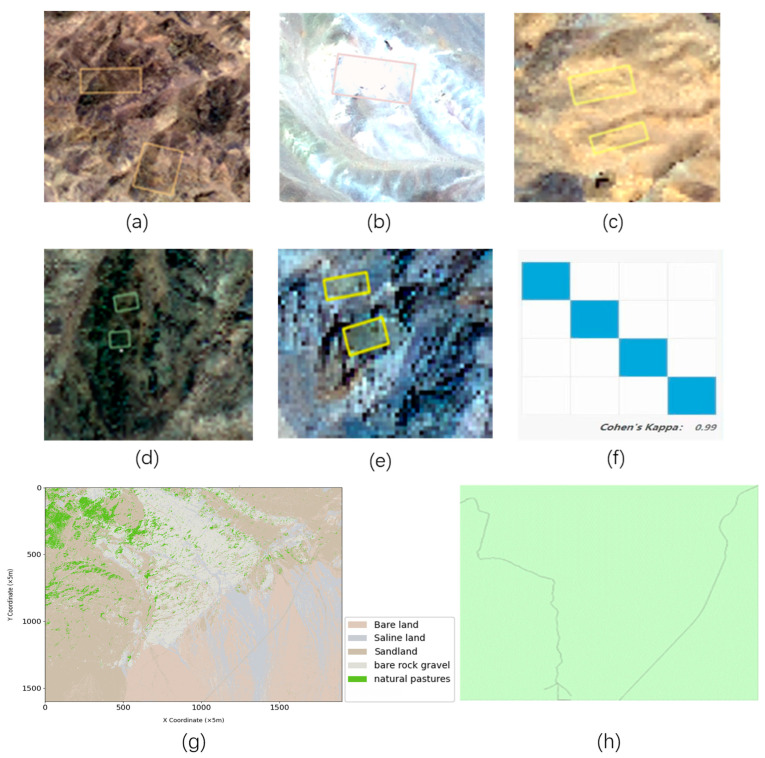
(**a**) is a bare land sample; (**b**) is a saline-alkali land sample; (**c**) is a sandy sample; (**d**) is a natural pasture sample; (**e**) is a sample of bare rock gravel; (**f**) is the Kappa value; (**g**) is the extraction map of supervised classified features; (**h**) is the road extraction map.

Based on the “Training Sample Manager” in GIS PRO software, we created training samples for supervised classification by drawing ROI vector files for different land cover types in the study area, including “bare land, sandy land, natural grassland, saline-alkali land, and bare rock gravel land.” Each land cover type exhibits unique spectral characteristics, providing significant distinguishing features for Support Vector Machine (SVM) classification. Saline-alkali land, due to its high salt deposits, typically appears bright white or gray, showing high reflectance. In contrast, sandy land is mainly yellow to light brown, reflecting its low vegetation coverage and mineral composition. Bare rock gravel land varies in color from gray to dark brown depending on the rock type, exhibiting high color saturation and contrast. Natural grassland is characterized by vibrant green, primarily determined by its rich chlorophyll content. Bare land color ranges from brown to red, depending on soil moisture and organic matter content. The training samples for different land covers are shown in Figure 5 a–e. After drawing the vector files for different land cover types, we selected the Support Vector Machine method for supervised classification. By analyzing and utilizing the unique color characteristics of the land, SVM effectively distinguishes between different land cover types, targeting pixels for classification within the study area. The accuracy of the SVM classification results was validated using Cohen’s Kappa coefficient, which yielded a Kappa value of 0.99, indicating high reliability and effectiveness of this method in land classification applications, as shown in Figure 5f.

The surface attributes are processed using GIS PRO software. High-quality images of off-road areas are categorized into the five main surface attribute classes above according to the classification standard of the Third National Land Survey. In addition, since the given off-road area contains several field roads, these roads are added as surface attributes for ground feature discrimination. Since field roads are formed after long-term crushing by off-road vehicles, the spectral properties of existing field roads and surrounding soil are similar in high-resolution images, and this similarity makes it difficult to distinguish roads and surrounding soil in image processing. Additionally, refined image analysis and processing techniques are needed to accurately recognize and extract road information. Therefore, a deep learning-based ground feature extraction method is used to extract roads in the field environment of the test area, as shown in Figure 5.

**Figure 6:**
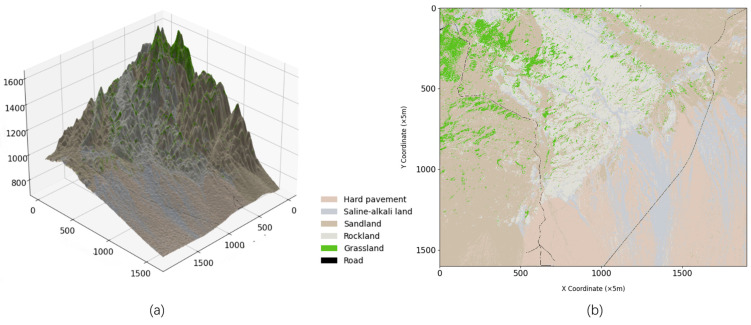
(**a**) is a three-dimensional off-road raster map that integrates features and elevation information; (**b**) is a flat off-road raster map that integrates features and elevation information.

To utilize the elevation information for mapping on a case-by-case basis, the area dominance method is used [36]; in this approach, rasters are associated with a single surface attribute. Notably, in the area dominance method, the attribute value of a raster is determined based on the attribute that accounts for the largest proportion of the raster area and the associated road information, elevation information, and feature attributes mentioned above. The information is integrated into a rasterized map to obtain a complete off-road raster map, as shown in Figure 6.

The main land surface properties influencing off-road vehicle use are the friction degree and the softness of the soil. The ground is divided into a bare soil environment and a vegetation cover environment, in which the soil type is mainly divided into sandy soil, clayey soil, and loam (the three types of soil are mainly classified based on the soil sand content). Surface cover includes vegetation, crops, etc. To quantify the effects of friction and softness on off-road vehicle use, the speed decay rate of off-road vehicles is calculated under different feature conditions. Additionally, a concise framework is established to assess how terrain friction and soil softness jointly affect the performance of off-road vehicles. In addition, the purpose of this experiment is to identify and quantify the extent to which these factors affect off-road vehicle travel to provide a scientific basis and reference for future off-road vehicle design and field navigation.

Several landform types in the test area are selected for analysis: asphalt road, grassland, saline-alkali land, sand, and gravel land. Notably, 100 m of relatively flat terrain was selected for each landform type to conduct analyses. Asphalt roads are smooth and continuous, and the road surface hardness is high, the rolling resistance is low, and road collapse during off-road vehicle use can be ignored; therefore, off-road vehicles traveling on asphalt roads are classified into the control group, and their speeds are used for reference.

An off-road vehicle travels at 1000 r/min (the speed of the car) under the same external conditions on each evaluated road type, and the travel time is recorded. The speed measured on the asphalt road ${\;V}_{0}$ is compared to the speed ${\;V}_{1}\;$ measured on other roads:

(4) $$\alpha \left(terrain\right)=\frac{V_{0}{-V}_{1}}{V_{0}}$$

α(terrain) is the attenuation rate of the vehicle travel speed for paths with different soil types; $V_{0}$ is the travel speed on asphalt; and $V_{1}$ is the speed of travel over different land types.

One hundred sets of repeated experiments are conducted in sand, grass, saline, and gravel conditions to calculate the rate of speed decay in reference to the asphalt travel speed, and finally, the calculated rate of speed decay is averaged to obtain a final estimate, as shown in Table 2.

## 3. Algorithm Construction and Description

### 3.1. A* Algorithm

The A* algorithm is a heuristic search method; notably, it is the most efficient direct search method for determining the shortest path in a static road network. The A* algorithm is a fusion algorithm based on a depth-first algorithm and a breadth-first algorithm. In the path planning process, the A* algorithm not only searches for the optimal path in the whole environment but also runs computationally faster than many other methods [44]. In this study, the algorithm is based on the following expression:

(5) $$f\left(n\right)=g\left(n\right)+h\left(n\right)$$

where $f\left(n\right)$ is an estimate of the cost of traveling from the initial state to the target state via state n, $g\left(n\right)$ is the actual cost of traveling from the initial state to state *n*, and h(n) is the estimated cost of traveling along the best path from state n to the goal state, also called the heuristic function. The value at the next reachable node $f\left(n\right)$ is calculated in real time for vehicles; thus, raster nodes are dynamically searched to find the minimum $f\left(n\right)$ and identify the path with the smallest cumulative value between the start point and a given destination.

In general, the heuristic function in the A* algorithm is used based on either the Euclidean distance or the Manhattan distance, depending on the conditions in different application scenarios. If a vehicle can move among four nodes at any given step, the Manhattan distance is chosen, and the standard heuristic function is based on the Manhattan distance, which considers the cost of movement; notably, the move associated with the minimum cost d is selected. If a vehicle can move at an arbitrary angle, i.e., there are eight nodes from which to choose, the Euclidean distance is more appropriate. To comply with the actual movement rules of off-road vehicles, the Euclidean distance should be used in the traditional A* algorithm to calculate the path cost, and the cost should be the sum of the parent node cost and the raster path cost. The specific cost function is as follows [45]:

(6) $$D=d\sqrt{{\left[n\cdot x-\left(n-1\right)\cdot x\right]}^{2}+{\left[n\cdot y-\left(n-1\right)\cdot y\right]}^{2}}$$

(7) $$h\left(n\right)=D\sqrt{{\left(n\cdot x-goal\cdot x\right)}^{2}+{\left(n\cdot y-goal\cdot y\right)}^{2}}$$

where  n·x and  n.y represent the *x* and *y* coordinates of the current node *n*, respectively. goal·x and  goal·y are the *x* and *y* coordinates of the target node, respectively; D represents the cost of moving from the current grid to a neighboring grid and is usually set to 1; and *d* represents the distance of between these grids.

### 3.2. Improvement of the A* Algorithm

#### 3.2.1. Data Structure Optimization

In the field of path planning, commonly used data structures include two-dimensional arrays and min-heaps, each with its own advantages and limitations. Two-dimensional arrays are simple and straightforward, well-suited for representing grid data of fixed size. They allow for efficient O(1) time complexity in node state lookup and updates. However, they lack flexibility when dealing with dynamic data and may consume significant space in large-scale grids. In contrast, min-heaps, which are a type of binary tree structure with priority characteristics, can quickly locate the node with the minimum cost function f value in O(1) time and perform insertions and deletions in O(log n) time. This efficient priority management makes min-heaps excel at handling dynamic node data, though they face challenges due to the higher complexity of data access.

This study innovatively combines two-dimensional arrays with min-heaps in path planning algorithms, leveraging the strengths of both while mitigating their respective weaknesses. Specifically, the two-dimensional array is used to store basic node information, such as g and h values, while the min-heap manages node priorities. This allows the algorithm to quickly identify the node with the minimum f value each time a node is expanded. Although this combination introduces additional complexity, such as increased overhead in data synchronization and structure management, the design results in significant optimization benefits. Firstly, in terms of time complexity, the traditional A* algorithm has an O(n) complexity for node selection operations. By introducing the min-heap, the improved algorithm optimizes this operation to O(1), greatly accelerating the speed of locating the optimal node. Additionally, the time complexity for insertion and deletion operations is improved from O(n) to O(log n), further enhancing the overall efficiency of the algorithm. Secondly, regarding space complexity, while combining the min-heap with the two-dimensional array does indeed consume additional storage space, the total space complexity of this combination remains at O(n), without a significant increase. Therefore, despite the added implementation complexity, this combination of data structures demonstrates excellent advantages in both time and space complexity, making it an effective and promising optimization strategy.

#### 3.2.2. Heuristic Function Design Considering Off-Road Factors

Considering the complex travel environment in the wilderness, existing wilderness roads are often characterized by relatively high accessibility. However, when off-road vehicles travel in complex wilderness environments, road factors are often ignored. To consider wilderness road factors, a new heuristic function incorporating these factors was integrated into path planning.

Since wilderness roads are often paths created by multiple passes of off-road vehicles and tend to have greater accessibility than their surroundings, the cost of access is lower for road grids than nonroad grids; consequently, a penalty factor is assigned to such grids to measure the cost of access, with the Euclidean distance as a benchmark:

(8) $$\left\{\begin{array}{c} D\left(node,goal\right)=\alpha *D+\beta *D\\ \alpha +\beta =1 \end{array}\right.$$

where D is the Euclidean distance, α is the penalty factor for a road raster, β is the penalty factor for a nonroad raster, and $D\left(node,goal\right)$ is the distance heuristic function based on road and nonroad factors.

The grid penalty coefficient is used to guide the algorithm to be dominated by road grids in the road finding process; based on this approach, the following heuristic function is set in this study:

(9) $$\left\{\begin{array}{c} H\left(node,goal\right)=D\left(node,goal\right)+T\left(node,goal\right)\\ D=\sqrt{{\left(goal_{x}-{node}_{x}\right)}^{2}+{\left(goal_{y}-{node}_{y}\right)}^{2}}\\ T\left(node,goal\right)=\frac{D\left(node,goal\right)}{v\left(1-\alpha \left(terrain\right)\right)} \end{array}\right.$$

where $T\left(node,goal\right)$ represents the time cost, v represents the off-road vehicle passing speed, and α(terrain) represents the speed decay rate of off-road vehicles under different conditions; the speed decay rate of off-road vehicles under different conditions and the feature weighting coefficients are shown in Table 2.

#### 3.2.3. Improvements to Neighborhood Searches

**Figure 7:**
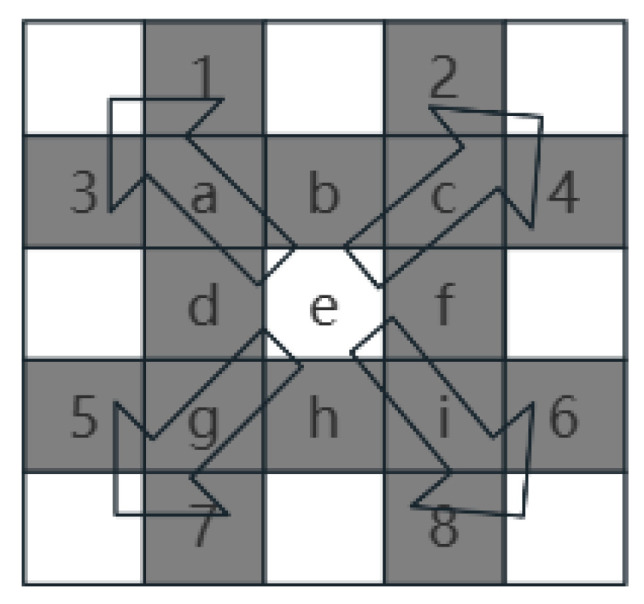
The 16-neighborhood search.

Currently, the commonly used path search methods of the A* algorithm are divided into 8 neighborhoods, 16 neighborhoods, and 32 neighborhoods. When searching for 16 neighborhoods and 32 neighborhoods, the algorithm not only has to judge whether the current node is passable, but also has to judge the connection with the previous node, which increases the complexity of the algorithm to a certain extent. Therefore, this article chooses to optimize and improve in the 16-neighborhood search direction. In addition to some traditional neighborhood search methods, Xie et al. [46] used a multi-directional search method based on circular radius to break through the direction limitations of the traditional A* algorithm by setting a reasonable search radius; Zhong et al. [47] set a specific threshold. Search, thereby reducing the search time and the number of nodes, and significantly improving the efficiency of path planning. This study will compare the performance of these two improvements to verify their advantages in path planning. The improvements of this study to the traditional 16-neighborhood are as follows:

As shown in Figure 7, the traditional A* algorithm searches all eight neighboring directions of each parent node; however, the 16-neighborhood algorithm adds a knight search on the basis of the eight-neighborhood search and expands the search scope, which inevitably results in some useless nodes being included in operations. Therefore, to avoid spending time evaluating irrelevant nodes, the optimization concept in this paper is as follows. According to the angle between the connecting line between the current node and the target point and the due east direction  θ, the search process is divided into six major categories, and then the search direction is assigned in real time according to different classifications. The specific classification rules are shown in Table 3.

In Table 3, all six classifications retain the important node directions and discard some unnecessary node directions, and the search direction is dynamically adjusted according to the θ value to avoid calculations involving useless nodes, which effectively improves the pathfinding efficiency.

## 4. Experiments and Results

In this study, the performance of the improved A* algorithm is evaluated based on the VScode2024 simulation platform. The experimental code is written in Python, the matplotlib library is used for graphing, and the heapq library is used to store the data in heap format. In the experiment, the start and end positions are entered, and the complete code is tested on a DELL OptiPlex 7000 computer with Intel Core i7-12700 @ 2.10 GHz to evaluate the performance of the improved A* algorithm.

The experiment consists of two parts: a performance test experiment of the improved neighborhood search and a performance test experiment of the improved heuristic function. In this test, the size of the simulated terrain area is 2000 × 1600, the raster resolution is 5 m, and the vehicle speed is set to 15 km/h. The coordinates of the starting point and the ending point are set to (100, 0) and (1500, 1500), respectively, and the multiple global path searches are carried out between these two points.

### 4.1. Improved Neighborhood Search Experiment

**Figure 8:**
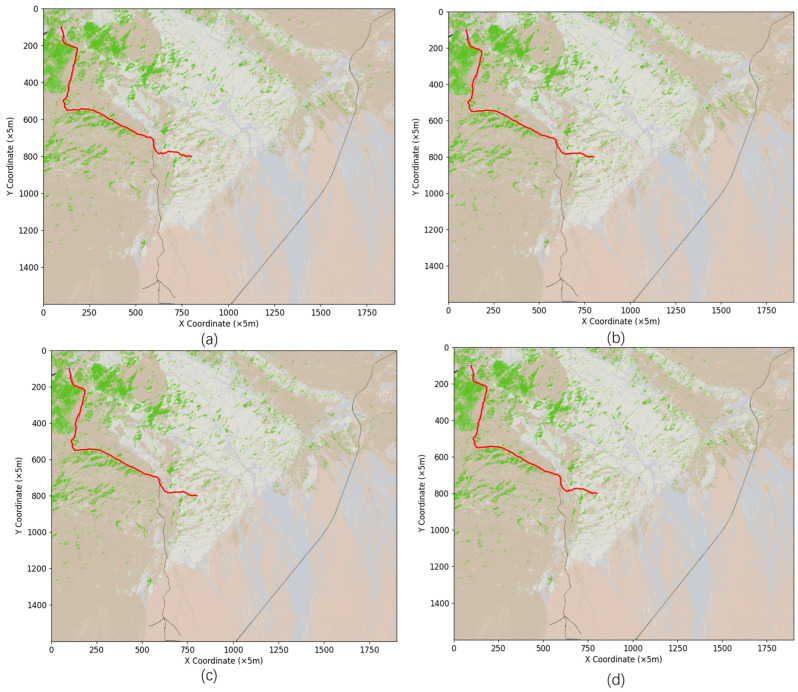
(**a**) is a path planning diagram of the improved heuristic function for 16 neighborhoods; (**b**) is a multi-directional path planning diagram; (**c**) is the path planning diagram of the optimization strategy search; (**d**) is the path planning map for improved neighborhood search.

To investigate the effectiveness of the dynamic search method in this study based on  θ different classifications, an experimental comparison between the traditional 16-neighborhood search and the improved 16-neighborhood search was conducted, as shown in Figure 8 and Table 4.

Based on the comparative analysis of the data in Table 4, simulation experiments were conducted for 16-neighborhood search, multi-directional search, optimized search strategy, and improved neighborhood search. The results indicate that while the differences in path distance among the four neighborhood search improvement methods are minimal, there are significant differences in the number of nodes and search efficiency. Compared to the traditional 16-neighborhood search, the improved neighborhood search reduced the number of nodes to 758, a decrease of 13.37%, and significantly improved search efficiency, with the shortest search time reaching 112.96 s, representing a 27.18% improvement in efficiency. Although the multi-directional search had the fewest nodes, its search efficiency was not as high as the other methods. On the other hand, the optimized search strategy had a number of nodes close to that of the traditional 16-neighborhood search but showed a significant improvement in search efficiency. These findings demonstrate that the dynamic 16-neighborhood search method proposed in this study has clear advantages in terms of efficiency and resource consumption, confirming the effectiveness of the search optimization strategy presented in this paper.

### 4.2. Comparative Experiments Regarding the Performance of Heuristic Functions Considering Off-Road Factors

**Figure 9:**
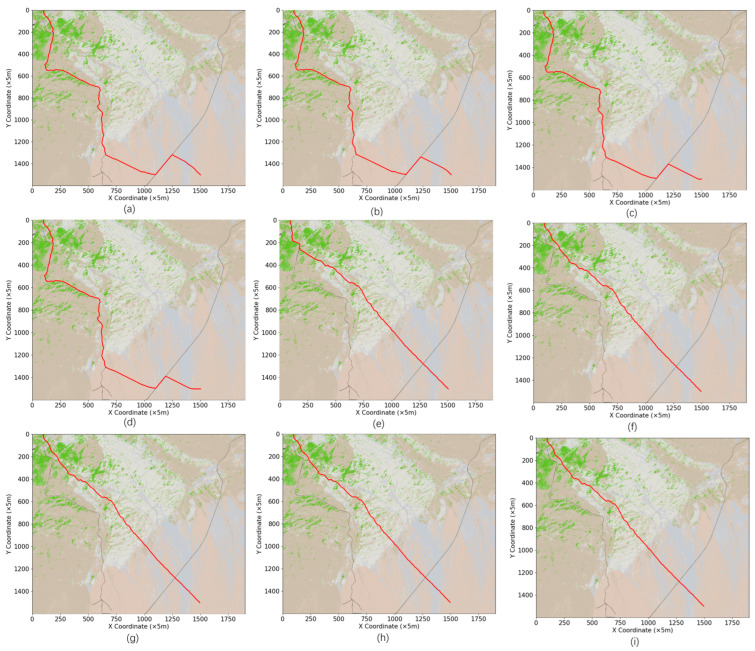
(**a**) α and β values are 0.1 and 0.9; (**b**) α and β values are 0.2 and 0.8; (**c**) α and β values are 0.3 and 0.7; (**d**) α and β values are 0.4 and 0.6; (**e**) α and β values are 0.5 and 0.5; (**f**) α and β values are 0.6 and 0.4; (**g**) α and β values are 0.7 and 0.3; (**h**) α and β values are 0.8 and 0.2; (**i**) α and β values are 0.9 and 0.1.

Based on the modified heuristic function, to determine the raster penalty coefficients in Equation (8), the optimal values of α and β were varied in simulation experiments, and the global path from the starting point (100, 0) to the end point (1500, 1500) was determined. The path diagram is shown in Figure 9, and the parameter results are given in Table 5.

Based on the above data, the optimal α and β combination is α=0.4 and β=0.6.

**Figure 10:**
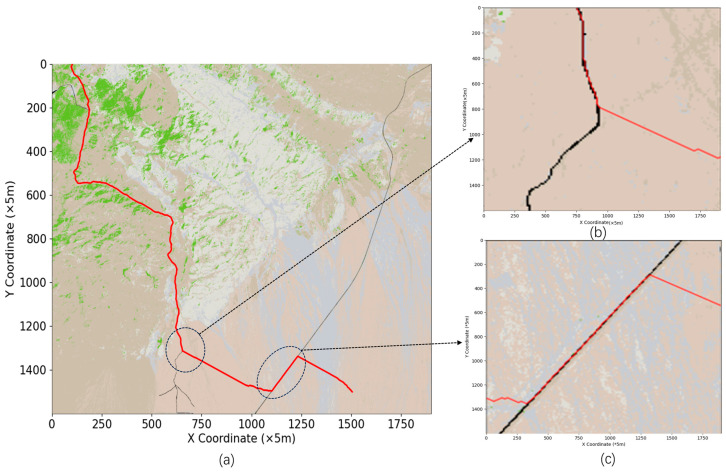
(**a**) is the path planning graph with α = 0.4 and β = 0.6; (**b**) is the path planning graph following unpaved roads; (**c**) is the excessive road dependence graph.

Setting the penalty coefficient for each raster aids in the selection of passable wilderness roads during path planning, as shown in Figure 10a,b. However, this path selection method may lead to road overdependence and increase the cost of path access, as shown in Figure 10c.

As shown in Figure 8a–d, the path planning process sets grid penalty coefficients α and β to favor the selection of highly traversable off-road paths. During path planning, the penalty coefficients make the algorithm more inclined to choose off-road routes with higher passability. This preference effectively avoids complex terrain and obstacles during the path planning process, resulting in relatively flat paths that can reduce travel time and vehicle wear and tear. This strategy is particularly suitable for scenarios with complex terrain and multiple possible routes, as it significantly enhances the passability of the planned paths. However, in Figure 10c, we can observe that this off-road prioritization strategy may lead to an over-reliance on roads. In certain situations, this dependency causes the path planning algorithm to overlook shorter or more direct routes, instead favoring longer but more passable roads. This blind reliance on roads ultimately increases the overall travel cost, leading to an increase in both the actual distance and time required for the path. Therefore, in practical path planning, balancing the passability of a path with its cost becomes a crucial issue. While choosing more passable roads can reduce immediate traversal difficulties, if not properly controlled, it can lead to an increase in the overall cost of the path. Consequently, in the design of the algorithm’s heuristic function, in addition to setting grid penalty coefficients, it is also necessary to introduce additional constraints or optimization strategies. These measures will help prevent blind dependence on roads, ensuring that while the path remains highly passable, the overall cost is optimized.

**Figure 11:**
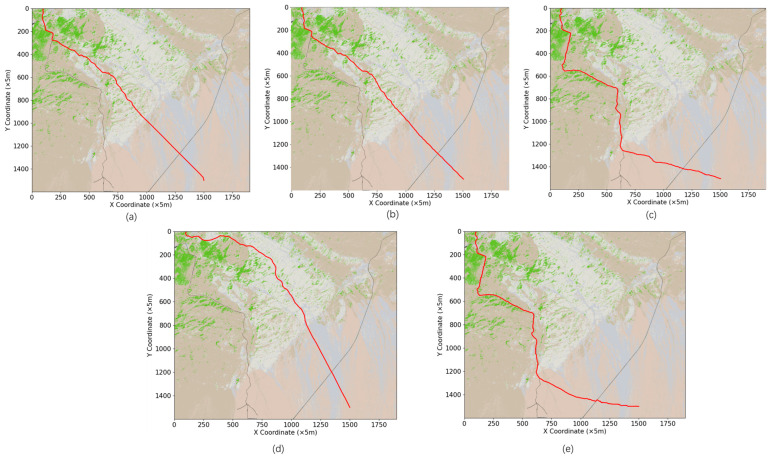
(**a**) is the path planned by the A* algorithm of the traditional 8-direction search; (**b**) is the path planned by the A* algorithm of the traditional 16-direction search; (**c**) is the path planned by the improved A* algorithm for multi-directional search; (**d**) The path planned for the improved A* algorithm of the traditional optimized search strategy; (**e**) improved the heuristic function and the path planned for searching for the neighborhood for this study.

In the above context, after adding distance and time factors to the heuristic function, the traditional A* algorithm and the improved algorithm are run with the same starting point and end point to carry out experiments. The results of the experiments are shown in Figure 11, and an algorithm performance comparison is shown in Table 6.

## 5. Discussion

In the process of off-road emergency rescue, the passability of paths and the complexity of terrain have considerable effects on the travel time of off-road vehicles. The accessibility of paths and changes in terrain lead to frequent changes in the direction of path searches and the expansion of the search range, issues that also exist in other path planning algorithms and are mitigated, to some extent, with the proposed approach. The improved A* algorithm in this study reduces the execution time of the algorithm by improving the search neighborhood and reduces the off-road emergency response time by searching for the most accessible paths. The improved A* algorithm in this paper mainly efficiency of path planning by identifying paths with high accessibility.

### 5.1. Optimal Values of α and β

In the process of improving the heuristic function, to focus on existing roads during path planning, the raster penalty coefficients α and β are used to classify road rasters and nonroad rasters, thus distinguishing the road environment from the off-road environment. In Figure 10, the horizontal axis represents the different α and β values, the left vertical axis denotes the path length, average elevation, and passage time, and the right vertical axis represents the average slope and search efficiency.

**Figure 12:**
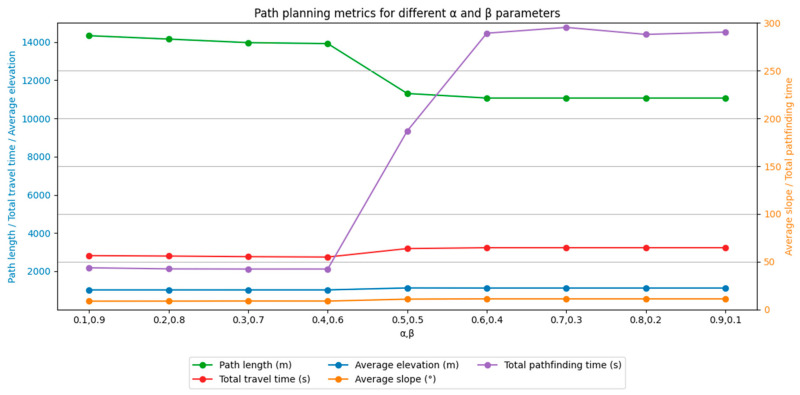
Path parameter comparison for different α and β values.

The results in Figure 12 are similar to those in Figure 9.

When  0.1≤α<0.5 (as in Figure 9a–d), as the α values increase, the paths follow existing roads in the wilderness environment, displaying strong road dependence. Notably, although the overall path complexity increases with increasing path length, complexity, and number of turns, the paths have a lower average elevation and lower average gradient and are more passable; thus, the travel and pathfinding times are minimized. This suggests that at lower α values, path accessibility and pathfinding efficiency are excellent despite the high path complexity.

When  0.5≤α≤0.9 (as in Figure 9e–i), as α increases, path selection gradually shifts away from the emphasis on existing roads. Notably, despite the reduction in path length, the mean elevation and mean gradient increase significantly, and the travel time and pathfinding time increase significantly; in particular, the search time displays an exponential increase. This suggests that at high α values, although the path length decreases, the travel time and search time significantly increase.

Considering the indicators together, the optimal α and β combination is α=0.4 and β=0.6. For this parameter combination, the path length is minimized (13,916.20 m), the average elevation and the average slope are kept at low levels (1024.65 m and 8.80 degrees), and the passage time and elapsed time are 2745.44 s and 42.37 s, respectively. In this case, the path distance is minimized, and the average elevation, average slope, and elapsed time of pathfinding are optimally balanced. Moreover, the paths obtained in this case are superior to those obtained with 0.5≤α≤0.9, even though the path length is greater, with more turns and higher complexity than the paths obtain in other cases.

### 5.2. Algorithm Performance Comparison

**Figure 13:**
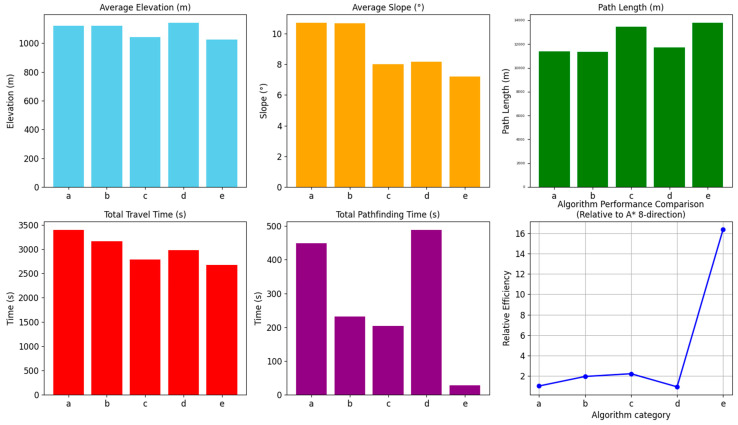
Comparison of path planning results for the improved algorithm and the traditional algorithm. a is the traditional A* algorithm (8 directions); b is the traditional A* algorithm (16 directions); c is the multi-direction method; d is the Optimized Strategy method; e is the Improved A* algorithm in this article.

With the above α and β parameters, the improved A* algorithm accounting for off-road factors is established with an improved heuristic function and a new search field approach, and this algorithm is compared with the traditional A* algorithm (8-direction and 16-direction) for path planning with the same starting point and end point. The average elevation, average gradient, path length, travel time, and search efficiency are shown in Figure 13.

Through the comparative analysis of the paths planned by different algorithms, it is evident that the improved A* algorithm demonstrates significant advantages across multiple dimensions. Compared to the traditional 8-direction search, the improved A* algorithm resulted in paths with an average elevation reduction of 97.61 m, an average slope decrease of 3.52°, a reduction in travel time by 12.059 min, and a remarkable 93.901% increase in search efficiency. This indicates that the improved algorithm can effectively reduce travel costs and significantly enhance path planning efficiency when dealing with complex terrains. Similarly, when compared to the traditional 16-direction search, the paths planned by the improved A* algorithm also show notable advantages, with an average elevation reduction of 95.81 m, an average slope decrease of 3.49°, a reduction in travel time by 8.11 min, and an 88.195% improvement in search efficiency. These results further validate the effectiveness and feasibility of the improved A* algorithm, which integrates road factors, in complex off-road terrain. Additionally, when comparing the performance of a multi-directional search and optimized search strategies, the improved A* algorithm proves superior in both search efficiency and the rationality of path selection, particularly in reducing travel time and enhancing search efficiency. The improved algorithm not only more accurately selects paths with lower elevations and slopes and higher passability, but also significantly shortens execution time, making it more adaptable and efficient for practical applications. In terms of relative efficiency (with traditional 8-direction search as the baseline), the line graph in Figure 13 shows that the efficiency of the improved A* algorithm is 16 times that of the traditional 8-direction search, far surpassing the relative efficiency of multi-directional search and optimized search strategies. This demonstrates that the improved A* algorithm not only has theoretical advantages but also significantly enhances path planning execution efficiency in practical applications, making it more suitable for complex off-road terrain scenarios. Thus, the improved heuristic function and neighborhood search in the proposed A* algorithm effectively consider the accessibility of the actual terrain surface, particularly in selecting existing off-road paths, enabling rapid and efficient application in various complex off-road terrain path planning scenarios.

In the same path planning task, the improved A* algorithm plans a longer path than does the traditional A* algorithm but displays better performance in terms of path passability, off-road vehicle travel time, and search efficiency. This result suggests that the improved algorithm yields better consistency and robustness. Generally, paths that have existed in the wild for a long time tend to be characterized by high passability, and the improved A* algorithm gives priority to finding these high-traversability paths during the path planning process. In contrast, the traditional A* algorithm generally uses a distance-based heuristic function in the path-finding process and prioritizes searching for paths that minimize the travel distance while ensuring obstacle avoidance. The improved A* algorithm in this study sacrifices path distance to a certain extent and prioritizes finding paths with maximum passability. This design choice improves travel efficiency and effectiveness.

## 6. Conclusions

In this paper, the effects of soil softness and terrain friction on off-road vehicle access are considered in the off-road path planning problem, accounting for terrain slope, feature access, and other factors that influence travel in wilderness scenarios. Through a combination of theoretical analysis and simulation experiments, a slope, feature access, and speed decay relational table was established to assess field access conditions for off-road vehicles. Considering that existing roads in the field are more accessible than nonroads for off-road vehicle access, raster penalty coefficients are set to guide the path search toward existing roads considering travel distance and time. The simulation results show that the number of nodes in the area of the improved search neighborhood is reduced by 16.784% and the search efficiency is improved by 39.42% compared to those of the traditional search algorithm; additionally, the improved path planning results are more efficient. Numerical experiments comparing the improved A* algorithm with the traditional A* algorithm indicate that the improved algorithm reduces the travel time of off-road vehicles by 21.298% and improves the search efficiency by 93.901%; moreover, the improved algorithm can produce optimal paths at a lower time cost, which is crucial for real-time off-road path planning.

Although the simulation experiments in this paper have verified the effectiveness of the proposed algorithm, we must recognize the differences between the simulated environment and the real world. Some variables in the simulation are strictly controlled, which, while useful for validating theoretical performance, cannot fully replicate the complexity and uncertainty of real-world scenarios. This study has primarily focused on path planning in off-road environments with unpaved roads. However, path planning in purely off-road environments has not been sufficiently addressed. Additionally, the algorithm’s handling of path planning under adverse weather conditions remains inadequate, which could affect its reliability in practical off-road vehicle applications. The algorithm’s innovativeness also needs to be further enhanced to cope with the more complex and variable real-world environments. Therefore, future research should focus on the following areas: First, more attention should be given to path planning in purely off-road environments to ensure that the algorithm can function effectively in complex terrains without roads. Second, consideration should be given to path planning under adverse weather conditions to improve the algorithm’s adaptability in extreme environments. Finally, the algorithm’s innovativeness should be continuously improved by exploring more advanced path planning methods, and its performance should be validated through field tests in real-world scenarios. This approach will not only help to verify the algorithm’s practical adaptability and robustness but will also provide empirical evidence for further optimization. These aspects will be the focus of our next research endeavors.

## Figures and Tables

**Table 1:** Division of slope intervals and setting of weights.

Hierarchy	Elevation	Slope Weight
0	0°~5°	1
1	5°~10°	0.8
2	10°~15°	0.6
3	15°~20°	0.4
4	20°~25°	0.2
5	25°~31°	0.1
6	≥31°	0

**Table 2:** Velocity attenuation of off-road vehicles given different features and feature weighting factors.

Topography	Velocity Decay	Feature Weighting Factor
Open road	0	1
Saline soil	0.228	0.8
Grasslands	0.337	0.7
Grave	0.565	0.4
Sandy beach or river bank	0.699	0.3

**Table 3:** Search direction rules.

Form	Angle (Between Two Intersecting Lines) θ	Deleted Nodes
I	[0°, 22.5°] ∪ (337.5°, 360°]	a, d, g, 3, 5
II	(22.5°, 90°]	d, g, h, 5, 7
III	(90°, 157.5°]	f, i, h, 6, 8
IV	(157.5°, 202.5°]	c, f, i, 4, 6
V	(202.5°, 270°]	b, c, f, 2, 4
VII	(270°, 337.5°]	a, b, d, 1, 3

**Table 4:** Comparison of search direction performance.

Neighborhood	Distance (m)	Number of Nodes	Search Time (s)
16-neighborhood	6540.29	875	88.83
Multi-directional search	6675.19	619	150.70
Optimize your search strategy	6542.65	873	112.96
Improved neighborhood	6820.19	758	24.68

**Table 5:** α and β parameter analysis table.

α and β	Average Altitude (m)	Average Slope (°)	Path Length (m)	Travel Time (s)	Search Efficiency (s)
0.1,0.9	1024.00	8.70	14,331.73	2819.00	43.65
0.2,0.8	1024.09	8.73	14,153.93	2796.06	42.49
0.3,0.7	1024.60	8.84	13,968.37	2764.76	42.36
0.4,0.6	1024.65	8.80	13,916.20	2745.44	42.37
0.5,0.5	1127.61	10.82	11,306.03	3188.21	186.93
0.6,0.4	1122.46	11.13	11,067.71	3232.78	289.23
0.7,0.3	1122.46	11.13	11,067.71	3232.78	295.32
0.8,0.2	1122.46	11.13	11,067.71	3232.78	287.98
0.9,0.1	1122.46	11.13	11,067.71	3232.78	290.39

**Table 6:** Comparison of the performance of the traditional A* algorithm and improved A* algorithm.

Algorithm Category	Average Altitude (m)	Average Slope (°)	Length of Path (m)	Travel Time (s)	Search Efficiency (s)
Traditional A* (8-way)	1122.51	10.71	11,380.43	3397.29	448.80
Traditional A* (16 directions)	1120.71	10.68	11,327.63	3160.34	231.86
Multi-directional search	1043.60	8.01	13,459.69	2784.9	203.35
Optimize your search strategy	1142.56	8.15	11,710.01	2980.76	489.19
Improved A*	1024.90	7.19	13,804.03	2673.71	27.37

## Data Availability

The data presented in this study are available upon reasonable request from the corresponding author.
